# Internet Resources for Gene Expression Analysis in *Arabidopsis thaliana*

**DOI:** 10.2174/138920208785699535

**Published:** 2008-09

**Authors:** Reinhard Hehl, Lorenz Bülow

**Affiliations:** Technische Universität Braunschweig, Institut für Genetik, Spielmannstr. 7, D-38106 Braunschweig, Germany

**Keywords:** Bioinformatics, databases, gene expression, plants, transcription, web-server.

## Abstract

The number of online databases and web-tools for gene expression analysis in *Arabidopsis thaliana* has increased tremendously during the last years. These resources permit the database-assisted identification of putative *cis*-regulatory DNA sequences, their binding proteins, and the determination of common *cis*-regulatory motifs in coregulated genes. DNA binding proteins may be predicted by the type of *cis*-regulatory motif. Further questions of combinatorial control based on the interaction of DNA binding proteins and the colocalization of *cis*-regulatory motifs can be addressed. The database-assisted spatial and temporal expression analysis of DNA binding proteins and their target genes may help to further refine experimental approaches. Signal transduction pathways upstream of regulated genes are not yet fully accessible in databases mainly because they need to be manually annotated. This review focuses on the use of the AthaMap and PathoPlant^®^ databases for gene expression regulation analysis and discusses similar and complementary online databases and web-tools. Online databases are helpful for the development of working hypothesis and for designing subsequent experiments.

## 1 INTRODUCTION

*Arabidopsis thaliana* is an important model organism for plant biologists. Its small genome is completely sequenced and contains only a low amount of repetitive DNA and a high gene density [1]. Furthermore, many mutants have been characterised phenotypically and insertion mutations for nearly all genes are available. An integrated information resource can be accessed at http://arabidopsis.org [2]. The large amount of information available for this model plant justifies its use for almost all basic biological questions.

All scientific questions that address developmental processes or biotic and abiotic signal response reactions focus on the understanding of gene expression regulation. There are three levels of regulation, pre-transcriptional, transcriptional and post-transcriptional. The pre-transcriptional level concerns chromatin-structure and remodelling. Transcriptional control is executed mainly by transcription factors (TFs) that recruit the transcriptional preinitiation complex to the promoter. A major aspect of post-transcriptional regulation is RNA stability affected by small RNAs. 

Out of these levels, transcriptional control is the most accessible level for database-assisted analysis [3]. Transcription factors bind to short sequence motifs, and families of TFs usually bind to similar sequences. In *Arabidopsis thaliana* more than 1500 TFs were initially identified which constitute at least 5% of all protein coding genes [4,5]. More recently, more than 2000 protein coding sequences comprising 68 families are predicted to be TFs [6]. 

Experimental data on these TFs varies significantly. While usually a few type members of each family have been extensively analysed, the function of all family members often remains unknown. The same applies for information on the binding site of these factors. For members of 25 TF families representative binding sites have been published and were annotated to databases [7-9]. 

A simple concept for transcriptional control is based on the presence of the binding site or *cis*-regulatory sequence in the promoters of genes which will then be a target site of a TF that regulates expression of the gene through DNA binding [3]. A next level of complexity is exerted by the combinatorial control of gene expression where TFs will bind after homo- or heterodimerization with other TFs [10]. 

Based on the occurrence or combination of *cis*-regulatory elements, predictions can be established about which TF family is involved in regulating transcription [3]. However, it is still a major challenge to predict particular TF family members that bind. For this, knowledge on a possible coexpression of a member of the putatively binding TF family with the target gene may be useful [11].

Often, single TFs are not sufficient for the regulation of gene expression [10]. To gain more insight into the complexity of expression control, it is helpful to learn if *cis*-regulatory elements recognized by known interacting TFs colocalize in their target genes [12]. Furthermore, additional information on protein-protein interactions may gain insight into upstream signal transduction pathways [13]. 

Another level of complexity that can be addressed with databases is the post-transcriptional control of gene expression. A large number of small RNAs have been cloned from *Arabidopsis thaliana* and the genomic identification of their target sequences may reveal which genes are subjected to small RNA-mediated degradation [14]. 

This review describes internet resources that are available for the study of gene expression regulation in *Arabidopsis thaliana*. It will focus on two databases, AthaMap and PathoPlant and the questions that can be addressed with them. The subjects discussed are schematically shown in the flow chart in Fig. (**1**). To address these subjects, also other online resources are available which are summarized in Table (**1**). 

## 2 THE ATHAMAP DATABASE

AthaMap is a database that generates a genome-wide map of putative transcription factor binding sites (TFBS) for *Arabidopsis thaliana*. AthaMap was initially developed by matrix-based sequence searches using alignment matrices derived from several binding sites of the same TF [7]. Subsequently, AthaMap was extended with functionally verified single TFBS [8]. Currently, the database contains 9.9 x 10^6^ predicted binding sites from 103 plant transcription factors [9]. Online AthaMap tools include a basic search function that requires a chromosomal position or a locus identifier (AGI). This results in a 1000 bp sequence display window with indicated putative binding sites and annotated gene structure. A colocalization function permits the identification of chromosomal positions of putative combinatorial elements [12]. Combinatorial elements were also precalculated and annotated to AthaMap based on TFs that are known to interact or that contain two DNA binding sites. A gene analysis function allows the identification of common or missing TFBS in a set of genes [9]. This function may be useful for the analysis of coregulated genes and for genes that are members of the same family. The data content and the use of the database is described online and in the respective publications. Furthermore, a short user manual has been published recently [15].

## 3 THE PATHOPLANT DATABASE

PathoPlant is a database on plant-pathogen interactions and signal transduction reactions [16]. The database contains microarray gene expression data from *Arabidopsis thaliana* subjected to pathogen infections, signal components, and elicitors [17]. Web-tools allow the identification of plant genes regulated by specific stimuli. Genes coregulated by up to three stimuli can be displayed as well. Furthermore, the web interface permits the submission of gene sets to be analysed for pathogen-responsive gene expression. A result table lists the stimuli that act either inducing or repressing on the respective genes. This is particularly useful if for example sets of genes have been identified previously which harbour similar TFBS. The search in PathoPlant can be restricted to certain induction factors to identify for example strongly up- or down-regulated genes. A resulting list of coregulated genes can directly be exported to the AthaMap database for analysis of common *cis*-regulatory elements.

## 4 WHICH TRANSCRIPTION FACTORS CAN BIND TO MY REGULATORY DNA SEQUENCE(S)?

**Fig. (1):**
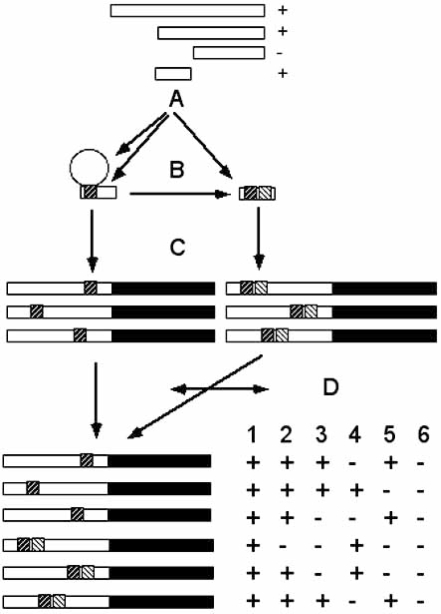
Database-based assisted analysis of gene expression. Internet resources permit the identification (**A**) of putative *cis*-regulatory sequences (hatched box) and binding transcription factors (circle) in an experimentally delineated regulatory region (+/-). These *cis*-regulatory sequences may be part of a known or predicted combinatorial element (**B**). Genes that harbor similar *cis*-regulatory sequences can also be determined (**C**). The identification of similar expression profiles in a set of genes with similar *cis*-regulatory sequences is illustrated for six different expression conditions (**D**). The double arrow indicates that the analysis can also start with a set of coregulated genes to identify similar *cis*-sequences.

The experimental analysis of gene expression may still include a classic deletion and reporter gene approach to identify a regulatory region in a gene. This may delineate a region that is essential for gene expression. A next step aims to predict the specific *cis*-regulatory sequence and/or transcription factor(s) that regulate(s) gene expression through this region (Fig. **1A**).

For this purpose the region of interest can be displayed in AthaMap and putative TFBS that occur in this region can be predicted [9]. This allows the development of a hypothesis which TFBS may be relevant for gene expression. For example, if a drought responsive element is detected in a drought responsive gene, further experiments may focus on this element. 

If the regulatory region is found in a promoter region, also other *A. thaliana* specific databases such as AGRIS, ATHENA, and ATTED-II (Table **1**) can be employed to predict *cis*-regulatory sequences [18-20]. Furthermore, the delineated regulatory sequence can also be submitted to the PLACE, PlantCARE, and TRANSFAC^®^ databases (Table **1**) to display either TFBS or *cis*-regulatory sequences that have been experimentally described before but for which no binding TF has been predicted [21-23]. 

## 5 IDENTIFICATON OF COMBINATORIAL REGULATORY ELEMENTS

In many cases a single TFBS does not cause a specific expression unless in combination with other regulatory elements. A database-assisted approach could answer the question if a regulatory sequence is part of a known combinatorial element (Fig. **1B**). Such combinatorial elements will also be displayed in AthaMap if they were previously annotated [12]. Furthermore, if several *cis*-regulatory elements or TFBS occur in the experimentally defined regulatory region it can be experimentally investigated if more than one is required for gene expression. 

A different approach may start with identifying putative interacting proteins using interactome databases [13,24]. If predicted interacting proteins are TFs, the gene under investigation may be analysed for the occurrence of TFBSs for this TF. If no binding site for this TF has previously been published, respective experiments to establish the TFBS for this factor may be carried out. 

## 6 DO OTHER GENES HARBOUR THE PREVIOUSLY PREDICTED TFBS?

If TFBS predicted by database-assisted analysis were experimentally confirmed to be relevant for gene expression, it may be interesting to find out if other genes harbour this combination of TFBS in a similar arrangement (Fig. 	**1C**). For this a web-tool to identify combinatorial elements in the AthaMap database can be employed [12]. It is possible to select two TFs that are relevant for gene expression on the web-server and to obtain a list of genes that harbour these TFBS in a user-defined arrangement. If only the occurrence of TFBS of one TF relative to other genes is of interest, AthaMap may not be employed yet. For this, ATHENA’s data mining tool permits the identification of genes that harbour selected TFBS (Table **1**). Furthermore, another tool to identify genomic positions of previously determined *cis*-regulatory sequences is PatMatch available at TAIR [25] (Table **1**).

## 7 IDENTIFICATION OF COREGULATED GENES WITH SIMILAR *CIS*-REGULATORY SEQUENCES

Another problem that can be addressed with web-resources is the identification of conditions under which genes with similar TFBS are coregulated (Fig. **1D**). A list of genes identified with AthaMap that harbour TFBS under investigation can be exported to the PathoPlant database to investigate if the genes are coregulated [9,17]. PathoPlant incorporates microarray expression data for pathogen and signal substance regulated genes. If additional gene expression profiles are of interest, other resources can also be used for this analysis. 

Several web-based services harbor gene expression data from *Arabidopsis thaliana* microarray experiments and allow recovery of information for individual genes or gene sets (Table **1**). These are for example TAIR [2,26], AtGenExpress [27,28], NASCArrays tools [29], Stanford Microarray Database (SMD) [30,31], Botany Array Resource now called Bio Array Resource (BAR) [32], Gene Expression Omnibus (GEO) [33], and Genevestigator [11]. Arabidopsis Coexpression Tool (ACT) [34,35], BAR [32], the Comprehensive Systems-Biology Database (CSB.DB) [36], and Genevestigator [11] allow comparative gene analysis to detect clusters of genes with similar expression patterns across selected or the complete set of stimuli. These tools start with a given gene of interest to determine similarities in expression patterns to other genes. 

## 8 IDENTIFICATION OF SIMILAR *CIS*-REGULATORY SEQUENCES IN COREGULATED GENES

A different approach to identify *cis*-regulatory elements starts with the identification of coregulated genes (Fig. **1D**). For example PathoPlant allows the identification of coregulated genes and the export of the gene list to AthaMap for subsequent TFBS identification [17]. The commercially available ExPlain™ Analysis Platform from Biobase GmbH (Table **1**) will identify combinatorial patterns of TFBS in a set of user-provided Arabidopsis genes. The analysis platform uses a matrix-based approach to identify TFBS in promoter regions and analyses the results with respect to specific patterns of TFBS that are overrepresented compared to a set of control genes [37].

Considering the fact that not all TFBS of predicted TFs are known, it may also be of interest to find patterns of conserved sequence motifs in a set of coregulated genes. For this purpose several online tools are available (Table **1**). For example the Regulatory Analysis Tools (RSA tools) offer the possibility to submit a set of promoter sequences to identify common motifs [38]. A similar way of analysis is offered by Promomer at the Bio Array Resource [32]. Promomer is a web tool to discover over-represented sequence motifs in regulatory regions from sets of *A. thaliana* genes. The tools available online may also be downloaded and implemented locally. For example the Binding-site Estimation Suite of Tools (BEST) includes four commonly used motif-finding programs: AlignACE, BioProspector, CONSENSUS and MEME and the optimization program BioOptimizer [39].

Since these programs often yield sequence motifs derived from similar sequences that are conserved in a set of sequences, it may be of interest to find out if the motifs have been identified previously as regulatory sequences or TFBS. For this, STAMP (Table **1**) may be used to query databases of known motifs with new motifs derived from similar sequences [40]. Since motifs from many plant-specific databases such as PLACE, PlantCARE, AGRIS, and AthaMap are available at STAMP it may be established that an identified motif has similarities to a known regulatory sequence or TFBS.

## 9 DISCUSSION AND FUTURE DEVELOPMENTS

A large number of internet resources are available for gene expression analysis in *Arabidopsis thaliana*. It is usually very important to use more than one resource if possible. First, each database has a different level of curation and contains different data. For example putative *cis*-regulatory sequences or TFBS can be determined with consensus sequences or can be detected with alignment matrices. They may either represent putative TFBS or sequence motifs conserved in coregulated genes. It is important to note that most of the putative *cis*-regulatory sequences or TFBS are probably not functional. This illustrates that database-assisted analysis is only a tool to refine or design experiments. Ideally a future map of TFBS contains also data from chromatin immunoprecipitations that reveal functional TFBS *in vivo* [41]. Furthermore, not all genes are transcriptionally regulated. It is estimated that, depending on the system analysed, as much as 50% of all genes may be post-transcriptionally regulated [42]. To learn which Arabidopsis genes are targets for small RNA-mediated mRNA degradation, the Arabidopsis Small RNA Project (ASRP) database can be employed (Table **1**) [43]. AthaMap was recently complemented with predicted target sites from an *Arabidopsis thaliana* small RNA transcriptome screening [14]. mRNA transcripts annotated in AthaMap were associated to these sites to identify putative post-transcriptionally regulated genes. The identification of such genes will refine the analysis of TF regulated gene expression (Bülow and Hehl, unpublished). 

With the application of massive parallel signature sequencing (MPSS) approaches, the analysis of gene expression is recently undergoing a revolutionary development [44]. With these developments we are not far from learning which genes are transcribed at a cellular level in time and space. The integration of such information in databases like Arabidopsis eFP Browser [45] and Arabidopsis Gene Family Profiler (aGFP) [46] will visualise the tissue- and state-specific expression of each gene during plant development (Table **1**). 

Ideally not only transcriptome data will be integrated into databases but also proteome data [47]. Protein synthesis is under translational control and, most importantly, proteins may move between cells [48,49]. There are already several protein databases. For example SUBA, a SUBcellular location database for Arabidopsis proteins (Table **1**) comprises 10 distinct subcellular locations, >6743 non-redundant proteins and represents the proteins encoded in the transcripts responsible for 51% of Arabidopsis expressed sequence tags [50]. Another example is ARAMEMNON, a database for Arabidopsis integral membrane proteins [51]. There is no integrated proteome database available for *Arabidopsis thaliana* yet, but a recent review on plant proteome analysis summarizes all Arabidopsis proteome articles published in 2006 [52].

An integrated gene expression database of the future will contain data on functional *in vivo* TFBS, transcription factor and target gene expression and on cellular localization of transcripts and proteins.

## Figures and Tables

**Table 1:** Alphabetical List of Names and Links of Web Resources Mentioned in the Text

Name	Link
ACT	www.arabidopsis.leeds.ac.uk/ACT
aGFP	http://agfp.ueb.cas.cz
AGRIS	http://arabidopsis.med.ohio-state.edu
Arabidopsis eFP	http://www.bar.utoronto.ca/
ARAMEMNON	http://aramemnon.botanik.uni-koeln.de/
ASRP	http://asrp.cgrb.oregonstate.edu/db/
AtGenExpress	http://www.weigelworld.org/resources/microarray/AtGenExpress/
AthaMap	http://www.athamap.de/
ATHENA	http://www.bioinformatics2.wsu.edu/cgi-bin/Athena/cgi/home.pl
ATTED-II	http://www.atted.bio.titech.ac.jp
BAR	http://bbc.botany.utoronto.ca
CSD.DB	http://csbdb.mpimp-golm.mpg.de/
DATF	http://datf.cbi.pku.edu.cn
Explain	http://www.biobase-international.com/
GEO	http://www.ncbi.nlm.nih.gov/geo
Genevestigator	https://www.genevestigator.ethz.ch
NASCArrays	http://affy.arabidopsis.info/
PatMatch	http://www.arabidopsis.org/cgi-bin/patmatch/nph-patmatch.pl
PathoPlant	http://www.pathoplant.de/
PLACE	http://www.dna.affrc.go.jp/PLACE/
PlantCARE	http://bioinformatics.psb.ugent.be/webtools/plantcare/html/
PlnTFDB	http://plntfdb.bio.uni-potsdam.de
RSA tools	http://rsat.ulb.ac.be/rsat
SMD	http://smd.stanford.edu
SUBA	http://www.plantenergy.uwa.edu.au/applications/suba2/index.php
TAIR	http://arabidopsis.org
TRANSFAC	http://www.gene-regulation.com/

## ABBREVIATIONS

TF: = Transcription factor
TFBS: = Transcription factor binding site
